# HIV in Children in a General Population Sample in East Zimbabwe: Prevalence, Causes and Effects

**DOI:** 10.1371/journal.pone.0113415

**Published:** 2014-11-20

**Authors:** Erica L. Pufall, Constance Nyamukapa, Jeffrey W. Eaton, Reggie Mutsindiri, Godwin Chawira, Shungu Munyati, Laura Robertson, Simon Gregson

**Affiliations:** 1 Department of Infectious Disease Epidemiology, Imperial College London, St. Mary's Campus, Norfolk Place, London, United Kingdom; 2 Biomedical Research and Training Institute, Avondale, Harare, Zimbabwe; Brighton and Sussex Medical School, United Kingdom

## Abstract

**Background:**

There are an estimated half-million children living with HIV in sub-Saharan Africa. The predominant source of infection is presumed to be perinatal mother-to-child transmission, but general population data about paediatric HIV are sparse. We characterise the epidemiology of HIV in children in sub-Saharan Africa by describing the prevalence, possible source of infection, and effects of paediatric HIV in a southern African population.

**Methods:**

From 2009 to 2011, we conducted a household-based survey of 3389 children (aged 2–14 years) in Manicaland, eastern Zimbabwe (response rate: 73.5%). Data about socio-demographic correlates of HIV, risk factors for infection, and effects on child health were analysed using multi-variable logistic regression. To assess the plausibility of mother-to-child transmission, child HIV infection was linked to maternal survival and HIV status using data from a 12-year adult HIV cohort.

**Results:**

HIV prevalence was (2.2%, 95% CI: 1.6–2.8%) and did not differ significantly by sex, socio-economic status, location, religion, or child age. Infected children were more likely to be underweight (19.6% versus 10.0%, p = 0.03) or stunted (39.1% versus 30.6%, p = 0.04) but did not report poorer physical or psychological ill-health. Where maternal data were available, reported mothers of 61/62 HIV-positive children were deceased or HIV-positive. Risk factors for other sources of infection were not associated with child HIV infection, including blood transfusion, vaccinations, caring for a sick relative, and sexual abuse. The observed flat age-pattern of HIV prevalence was consistent with UNAIDS estimates which assumes perinatal mother-to-child transmission, although modelled prevalence was higher than observed prevalence. Only 19/73 HIV-positive children (26.0%) were diagnosed, but, of these, 17 were on antiretroviral therapy.

**Conclusions:**

Childhood HIV infection likely arises predominantly from mother-to-child transmission and is associated with poorer physical development. Overall antiretroviral therapy uptake was low, with the primary barrier to treatment appearing to be lack of diagnosis.

## Introduction

In 2012, it was estimated that over 85% of children who became infected with HIV were living in sub-Saharan Africa (SSA) [1]. However, general population data about epidemiology and health effects of paediatric HIV in SSA are sparse. The most common data about HIV prevalence in SSA, Demographic and Health Surveys (DHS) and community-based cohort studies, have typically only included persons over age 15 years. As a result, estimates for HIV in children are generally extrapolated from data about pregnant women using mathematical models [2]. In Zimbabwe, UNAIDS estimated that 2.8% (95% CI: 1.6–3.7%) of children 0–14 were HIV-positive in 2012 [2], [3]. Direct empirical data about the epidemiology, sources and impacts of HIV in children will improve confidence in estimates and ensure that health and social care systems are able to meet the needs of infected children.

Most infected children are believed to have acquired HIV perinatally from their HIV-positive mothers. Untreated HIV infection in infants is typically characterised by rapid disease progression and death at a median of two years of age or less, with survival depending at what stage (*e.g.* perinatally, breastfeeding) the infant becomes vertically infected [4], [5], but it is estimated that perhaps up to a third of vertically infected children survive into adolescence [6]–[8] and clinical reports have provided evidence of non-sexually acquired infections in adolescents [9]–[12]. However, debate continues as to whether or not these children are actually long-term survivors of mother-to-child transmission (MTCT) or have acquired HIV horizontally. Other studies have reported instances of horizontal HIV transmission in children [13]–[15]; however, these studies used non-representative samples or were conducted in highly localised areas.

In this study, we aim to: (i) describe patterns of HIV infection in a representative general population sample of children aged 2–14 years in a large-scale generalised HIV epidemic in rural areas of eastern Zimbabwe; (ii) investigate possible sources of horizontal HIV transmission in childhood; (iii) assess whether the observed age-pattern of HIV-positive children is consistent with that expected from survival of children infected from MTCT (given recent trends in adult prevalence and prevention of mother-to-child transmission (PMTCT) program scale-up); (iv) assess the impact of HIV on children's mental and physical health and nutritional status; and (v) investigate the levels and determinants of antiretroviral treatment (ART) coverage in children.

## Methods

### Study Population and Data Collection

The Manicaland HIV/STD Prevention Project is a population-based, open cohort study in eastern Zimbabwe [16]–[18]. Each round of the survey involves a census of all households in the 12 study sites (4 subsistence farming areas; 4 large-scale commercial estates; 2 small towns; and 2 roadside settlements), followed by interviews with individual household members and collection of dried blood spot samples for HIV testing.

In the most recent round (2009–2011) of the Manicaland survey, all children (aged 2–14 years) in a randomly selected 1/3 of households were invited to participate in an investigation of HIV prevalence amongst children. Children were interviewed about their welfare, health, and healthcare using a structured questionnaire. Children under seven answered with assistance from their primary caregiver. Questions on HIV testing and knowledge of HIV status were addressed to the child's primary caregiver in the presence of the child if he or she was over the age of seven. Additionally, the questionnaire was administered by a nurse who had HIV Testing and Counselling certificates, which include training in how to respond if a child becomes distressed. More sensitive questions were asked only of older children and were answered without their caregiver being present: children aged 7–14 years were asked questions on sexual abuse and on psychological health. If a child reported abuse then the interviewer notified the supervising nurse who would subsequently investigate in the company of a social worker. The information from the supervisor and social worker was then fed back to the Child Protection committee in the study area. All maternal data (religion and HIV status) were collected in the general (adult) survey and linked to child data based on children reporting who their biological mother was, and confirmed through fertility histories and the household roster. In cases where a link could not be made, or if the child was a maternal orphan, maternal data was coded as missing. Dried blood spot samples were collected and tested for HIV in an offsite laboratory using the COMBAIDS-RS HIV 1+2 Immunodot Assay (Span Diagnostics, India); for cases in which the child tested HIV-positive but had an uninfected mother, the HIV test results were confirmed using Vironostika HIV Uni-form II Plus O (Biomérieux, France) ELISA tests. Data used in the manuscript are provided in the supporting information file Dataset S1.

### Ethics Statement

Ethical approval for the Manicaland HIV/STD Prevention Project was obtained from the Research Council of Zimbabwe (Number 02187), the Biomedical Research and Training Institute Zimbabwe's institutional review board (Number AP6/97), and the Imperial College London Research Ethics Committee (Number ICREC 9_3_13). Written informed consent was obtained prior to survey participation from each child's primary caregiver. In addition, children aged 7–14 years provided verbal or written assent, respectively. Participants and guardians were informed that, at any point, they could refuse to answer a question or decline to continue the interview.

### Data Analysis

In this analysis of children aged 2–14 years old, we tested for associations of HIV infection with socio-demographic characteristics (sex, age-group, household socio-economic status (SES), community type, and mother's religion) using logistic regression. Socio-economic status was measured using a summed asset-based wealth index developed for the study population in Manicaland [19]. The mother's self-reported religious affiliation was classified into “Christian”, “Traditional”, “Spiritual”, “Other”, or “none”, as in previous analyses of Manicaland data [20].

To test the hypothesis that HIV infection in children occurs primarily through MTCT, where available, we examined maternal survival/infection status (deceased, alive and HIV-negative, alive and HIV-positive, alive with unknown HIV status) by child HIV status to establish the plausibility of vertical HIV transmission. The odds ratios of being a maternal orphan and of being a maternal orphan or having an HIV-positive mother amongst infected and uninfected children were evaluated using a one-sided Fisher's exact test. We tested for associations between HIV infection and risk factors for horizontal HIV transmission, which included blood transfusion, vaccination, non-vaccination medical injections, breastfed by a non-biological mother, cared for a sick relative, and sexual abuse.

To assess whether the observed age-pattern of HIV prevalence in children is consistent with that which would be expected in Zimbabwe if infections were due to mother-to-child transmission, we compared the age-specific HIV prevalence data to national estimates of child HIV prevalence reported by UNAIDS [1]. These estimates are derived using the Spectrum model [21]–[23], which assumes MTCT is the source of paediatric HIV and reflects the declining trends in HIV prevalence recorded in pregnant women, rates of mother-to-child transmission, patterns of paediatric survival by time of infection, national data on PMTCT and anti-retroviral therapy (ART) scale-up, and effectiveness of PMTCT regimens [4]. The Spectrum file that we used in this analysis can be downloaded from http://apps.unaids.org/spectrum/.

The impact of HIV on measures of physical and mental health was evaluated using linear (continuous outcomes) or logistic (binary outcomes) regression, adjusting for age. Z-scores for height- and weight-for-age and weight-for-height were calculated using WHO child growth standards [24], [25]. Z-scores below -2 were considered to indicate stunting (low height-for-age), being underweight (low weight-for-age) and wasting (low weight-for-height). Comparisons were made for stunting and being underweight for all children, while wasting was only compared in children aged 2–5 years, as these were the ages for which reference data were available [24]. Psychological wellbeing scores were calculated in children aged 7–14 years using principal components analysis of psychological distress measures as described by Nyamukapa *et al*., 2010 [26]. All analyses were conducted in Stata version 12.1 (StataCorp LP, USA).

## Results

### Demographic Profile of Infected Children

Four thousand six hundred and eleven children aged 2–14 years were enumerated and selected for inclusion in the study, of which 3389 (73.5%) completed the survey and gave a dried blood spot for HIV testing. Children who did not complete the survey did not have significantly different age or gender distributions and their household of residence did not have significantly different mean SESs than those who completed the questionnaire (all p>0.05). Reasons given for non-response included: away from home for work (7.4%), away from home for school (7.0%), another reason for being away from home (67.1%), whereabouts unknown (1.1%), refused (12.3%), and other (5.0%). Seventy-three (2.2%, 95% CI: 1.7–2.6%) were HIV-positive. Prevalence was 1.6% (11/688), 2.5% (33/1296), and 1.8% (25/1405) among children aged 2–4 years, 5–9 years, and 10–14 years, respectively. Demographic characteristics of children aged 2–14 years by HIV status are presented in Table 1. HIV prevalence did not differ significantly (p<0.05) by sex, age-group, or any other demographic characteristics (household SES, community type, and maternal religion).

**Table 1:** Association between demographic characteristics and HIV infection in children.

Category	Sub-category	HIV+	N in Sub-category	OR (95% CI)†	*p-*value‡
Gender	Male	2.34%	1712	Referent	0.21
	Female	1.73%	1677	0.75 (0.46–1.21)	
Age Group	2–4	1.60%	688	Referent	0.27
	5–9	2.54%	1296	1.61 (0.81–3.20)	
	10–14	1.78%	1405	1.11 (0.55–2.28)	
Household SES	Poorest quintile	1.75%	688	Referent	0.33
	Second quintile	2.38%	632	1.37 (0.64–2.95)	
	Middle quintile	0.70%	143	0.40 (0.05–3.07)	
	Fourth quintile	2.70%	1063	1.58 (0.80–3.13)	
	Least poor quintile	1.85%	863	1.06 (0.50–2.26)	
Community type	Subsistence farming	2.44%	1391	Referent	0.38
	Roadside trading	1.93%	671	0.79 (0.41–1.50)	
	Agricultural estate	1.36%	811	0.54 (0.27–1.08)	
	Commercial centre	2.13%	516	0.87 (0.44–1.73)	
Mother's religion^a^	Christian	1.44%	967	Referent	0.95
	Traditional	0%	13	N/A	
	Spiritual	1.53%	849	1.02 (0.48–2.19)	
	Other	1.69%	301	1.15 (0.41–3.21)	
	None	0%	67	N/A	

† Unadjusted odds ratio.

‡ Fisher's exact test for difference of proportions.

a Total respondents (N = 2206) is lower than other categories due to maternal orphans (n = 348), unlinked records (n = 722) and question non-response (n = 113).

### Sources of HIV Infection

All but one HIV-positive child were either maternal orphans or had an HIV-positive surviving mother, consistent with the primary source of childhood infection being MTCT (Table 2). HIV-positive children were significantly more likely to be a maternal orphan (OR: 6.56, 95% CI: 4.03–10.66) and/or have an HIV-positive mother (OR: 76.03, 95% CI: 18.54–311.79)) than HIV-negative children. The one child who was HIV-positive but for whom the woman identified as his biological mother was HIV-negative (Table 2) was a three year-old male reported to be living with both biological parents. He did not report any of the risk factors for non-sexual horizontal transmission (blood transfusions, non-medical injections, breastfeeding by a non-biological mother, caring for a sick relative, or child abuse). Overall, 26.9% (902/3360) of participants who answered the survey questions reported any of the selected risk factors for horizontal HIV transmission (Table 3), excluding vaccination-related injections, of which 99.7% (3364/3374) of children reported having had. Item non-response ranged from 0.1% (ever cared for a sick relative) to 27.1% (ever had a blood transfusion) and did not differ between HIV-negative and HIV-positive children (all p>0.05). HIV-positive children were significantly more likely to report non-vaccination injections than HIV-negative children (41.1% vs. 26.2%, p = 0.01). Otherwise, no significant differences in reporting of risk factors (blood transfusions, breastfeeding by a non-biological mother, caring for a sick relative, child abuse or sexual activity) were found between HIV-positive and uninfected children.

**Table 2:** Maternal mortality and HIV status in children.

	Child HIV status	
Maternal status	HIV- (N = 3316)	HIV+ (N = 73)
Mother deceased	318 (9.59%)	30 (41.09%)
Mother alive, HIV+	390 (11.76%)	31 (42.47%)
Mother alive, HIV-	1765 (53.23%)	1 (1.37%)
Mother alive, unknown HIV status	843 (25.42%)	11 (15.07%)

**Table 3:** Association between HIV status and potential horizontal risk factors for HIV.

Horizontal risk factors	Exposure	N	HIV+%	AOR (95% CI) †	*p*-value‡
Ever had a blood transfusion	No	3,355	2.15%	Referent	0.14
	Yes	7	14.3%	6.51 (0.80–53.34)	
Lifetime number of non-vaccination injections	0	2491	1.73%	Referent	0.004
	>0	873	3.44%	2.19 (1.23–3.89)	
Received tuberculosis, polio, measles, and/or diphtheria vaccination	No	10	0%	Referent	0.81
	Yes	3364	2.11%	N/A	
Breastfed by non-biological mother	No	3359	2.14%	Referent	0.71
	Yes	16	0%	N/A	
Cared for a sick relative (ages 6–14)	No	2403	2.29%	Referent	0.45
	Yes	35	0%	N/A	
Ever been sexually abused (ages 7–14)	No	2180	2.25%	Referent	N/A
	Yes	4	0%	N/A	

† Adjusted odds ratio; adjusted for age, gender, SES, and site type.

‡ Fisher's exact test for difference of proportions.

NB: Different Ns are due to different question non-response rates.

### Comparison of Observed Age-Specific HIV Prevalence with National Estimates from the Spectrum Model

**Figure 1:**
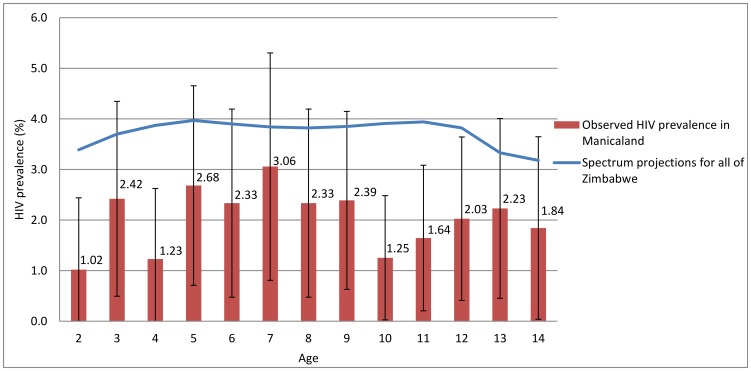
Comparison of observed HIV prevalence by age in Manicaland, with 95% confidence intervals, to the Zimbabwe national HIV estimates from UNAIDS and the Zimbabwe Ministry of Health and Child Welfare.

HIV prevalence in children aged 2–14 observed in Manicaland was lower than the national estimates for Zimbabwe as a whole in 2010 from the Spectrum model (3.6%). However, the age-pattern of HIV prevalence amongst children observed in the data was consistent with the model estimates (Figure 1). This suggests that the age-patterns of HIV in Manicaland are in line with what would be expected if MTCT were the main source of child infections, accounting for the declining trend in adult HIV prevalence and the scale-up of PMTCT programmes in Zimbabwe.

### HIV Status and Child Health

HIV-positive children were significantly more likely to be underweight (low weight-for-age) (AOR: 2.20; 95% CI: 1.08–4.47) and stunted (AOR: 1.69; 95% CI: 1.02–2.81) than HIV-negative children, but were not more likely to be wasted (low weight-for-height) (AOR: 0.43; 95% CI: 0.05–3.34) or to report a recent illness (AOR: 1.31; 95% CI: 0.64–2.66) (Table 4). HIV status was also not associated with psychological wellbeing (Coefficient: −0.06; 95% CI: −0.21 – +0.09) (Table 4).

**Table 4:** Effects of HIV status on physical and mental health outcomes in children and adolescents.

Health outcome	HIV status	N	%	AOR (95% CI)†	*p-value*
Ill in last two weeks	HIV−	3320	11.55%	Referent	0.11
	HIV+	69	19.61%	1.31 (0.64–2.66)	
Low height-for-age	HIV−	3222	30.60%	Referent	0.04
	HIV+	69	39.13%	1.69 (1.02–2.81)	
Low weight-for-age	HIV−	2225	9.97%	Referent	0.03
	HIV+	51	19.61%	2.20 (1.08–4.47)	
Low weight-for-height‡	HIV−	833	11.64%	Referent	0.42
	HIV+	16	6.25%	0.43 (0.05–3.34)	
Psychological wellbeing score^a^	HIV−	2385	0.01	Referent	0.45
	HIV+	55	−0.05	−0.06 (−0.21–+0.09)	

† Adjusted odds ratio; adjusted for age, gender, SES, and community type.

‡ Children 2–5 only.

a Mean and change in score between HIV− and HIV+; ages 6–14 only.

Of the 73 HIV-positive children, 26.0% (19/73) reported that they knew their HIV status. Of the HIV-negative children, 114/3309 (3.5%) had had an HIV test, significantly less than reported testing prevalence in HIV-positive children (p<0.001). Children of mothers who reported that they knew they were HIV-positive were 5.17 times (95% CI: 2.27–11.76) more likely to have had an HIV test and know the result than children of mothers who self-reported as HIV-negative. Knowledge of HIV status was not associated with psychological wellbeing score for HIV-positive children (change in psychological wellbeing score: +0.01; 95% CI: −0.131– +0.33). All but two of the children (17/19) who were aware of their HIV-positive status reported taking drugs that stop HIV from causing AIDS (*i.e.* were on anti-retroviral therapy (ART)). Despite high ART coverage when HIV status is known, overall, less than a quarter (23.3%, 17/73) of the HIV-positive children was receiving ART.

## Discussion

This study describes the prevalence and consequences of HIV in children living in a rural area of southern Africa. In eastern Zimbabwe, from 2009–2011, 2.2% (95% CI: 1.7–2.6%) of children aged 2–14 years tested positive for HIV, at a time when HIV prevalence was 11% and 17%, respectively, amongst male and female adults (15–54 years) in the same population. This estimate, from a representative general-population sample, is lower than those from a sample of children in 2005 in Chimanimani district in southern Manicaland, where HIV prevalence was 3.2% (41/1290) in children aged 2–14 years [27]. Such a reduction between 2005 and 2010 is expected based on the decline in adult HIV prevalence and the increase in PMTCT coverage since 2005. A study conducted with 4,386 primary school children in Harare in 2010 found an HIV prevalence of 2.7% (95% CI: 2.2–3.1%) in children aged 6–13 years [28], which is close to the prevalence of 2.2% found in our study for a slightly different age-group in a different region. As was the case in the Chimanimani study [27] and in studies in similar age-groups elsewhere in SSA [29]–[31], including a large national population survey in South Africa conducted in 2008 [32], we found no significant differences in HIV prevalence with respect to sex or age.

Our finding of a relatively even pattern of HIV prevalence by age is consistent with official national estimates derived from the Spectrum model. Survival data for children infected with HIV through MTCT suggest high mortality [7] and, in a stable epidemic with little horizontal transmission and no PMTCT intervention, HIV prevalence will decline as children age into adolescence. However, the decline in HIV prevalence in pregnant women since the late 1990s (from 25.7% in 2002 to 16.1% 2009 [33]) and the scale-up of PMTCT services from the mid-2000s explain reduced prevalence in younger children to the levels observed in the current study. The pattern of HIV prevalence we saw with age is also consistent with that reported by Eaton et al. in 15–17 year-olds in the same population at different time points (2009–2011 here and 2006–2008 in Eaton et al.) [12]. That is, it supports the hypothesis that MTCT is the main source of HIV infection in children and adolescents in this population.

Our data further confirm the belief that MTCT is the primary mode of HIV transmission in children in eastern Zimbabwe [12]. Mothers of HIV-infected children were significantly more likely than mothers of uninfected children to be deceased or HIV-positive. One child, for whom we could not identify a plausible source of infection, did not report any vertical, sexual, or other horizontal risk factors for transmission. Exposure to potential modes of transmission may have been under-reported and data were not collected on all possible sources of infection, such as scarification and hospital and dental visits, which have previously been identified as sources of HIV in children [13]–[15]. Sexual abuse has also been identified as a potential mode of HIV acquisition in select cases in children [34]–[39], however, due to ethical reasons, little research has been conducted into the proportion of children infected with HIV through sexual abuse, even though sexual abuse has been reported to be common in South Africa [40], [41]. While we cannot be certain about the accuracy of reporting about the child's biological mother, without biological tests, for which this study did not have consent, the identification of the biological mother was consistent with information reported on the household roster and the child was named on the mother's fertility history.

The need to understand HIV infection in children is particularly important given that HIV is increasingly becoming a cause of hospitalisation amongst adolescents in SSA [11] and this trend is likely to continue as more HIV-positive children age into adolescence. We found that HIV-positive children were significantly more likely to report non-vaccination medical injections than HIV-negative individuals, most likely because HIV-positive children are more likely to seek healthcare for managing their infection or to treat HIV-associated illnesses [11]. Thus, this association should therefore not be misconstrued as evidence for medical injections as a source of horizontal transmission, particularly as 27 of the 30 (90%) of the HIV-positive children who reported having had medical injections also reported being a maternal orphan or having a mother who was HIV-positive. Stunting and wasting in HIV-positive children, as well as being underweight, have been reported previously in SSA [11], [42], [43]. We found HIV-positive children to be significantly more likely to be underweight and stunted, indicative of the long-term harm of HIV infection on health and nutritional status. Perhaps unsurprisingly, we did not find a significant relationship with wasting, which measures recent severe weight loss, often associated with acute starvation and severe disease.

There are few data from SSA on the psychological manifestations of HIV infection in children, although studies from developed countries report significantly higher incidence of psychiatric admissions for HIV-positive children than HIV-negative children, with knowledge of HIV status increasing the risk of admission [44]. A previous study in Zimbabwe found that 56% of HIV-positive adolescents reported psychosocial problems, but that these problems were not common in younger children [45]. These data, however, were collected from children and adolescents visiting facilities offering HIV care services, and many respondents were already presenting with AIDS-related illnesses. Because the results were from a clinical study and were not compared to an HIV-negative population, it is not possible to conclude that there was a significant increase in psychosocial distress based on HIV status. Although we found no significant association between HIV status and psychological wellbeing, the lack of data from SSA in this area and the findings of Ferrand *et al*. (2010) [45] suggest that this is an important topic for future investigation.

Only a quarter of HIV-positive children in our study were aware of their HIV status. Across southern and eastern Africa, ART coverage among children is a major problem and high priority for many governments as it continues to lag behind adult coverage (33% versus 65% in the 22 priority countries, of which 21 are in Africa) [46]. So long as they remain undiagnosed, and therefore untreated, HIV-positive children are at higher risk for AIDS-related illnesses and early mortality. In the longer term, knowledge of HIV status is important to mitigate the risk of passing the infection on to sexual partners. Despite the low coverage of HIV testing, we found that if children or their guardians were aware of the child's HIV-positive status there was a 90% chance that the child would be on ART. This suggests that, despite the fear, potential stigma and associated costs, a positive diagnosis does result in the initiation of treatment. One way to help increase treatment amongst children might be to increase HIV-testing of women, as we found that when a mother knew herself to be HIV-positive, her child was significantly more likely to have had an HIV test themselves – suggesting that getting more mothers diagnosed through PMTCT programs will improve child testing. Other possible ways to increase infant diagnosis of HIV in this population are the new point-of-care tests currently being developed. New tests include a rapid p24 antigen test and a nucleic-acid amplification test, both of which can be performed in under half an hour [47]. Currently, many infants go undiagnosed due to the long turnaround times or poor infrastructure associated with dried blood spot sample testing [48]. Early infant HIV diagnosis is important, as the high ART coverage when children are aware of their status implies that lack of knowledge of HIV status is a contributing factor to the low coverage of ART in children that has been noted in Zimbabwe (39.5% according to the 2011 national estimates [49]) and more broadly in most of sub-Saharan Africa.

## Conclusion

These findings provide evidence that MTCT is the principal source of HIV infection in children in southern Africa and that current initiatives to increase the availability and effectiveness of PMTCT should result in reductions in HIV prevalence in children over time. Effort should be made to encourage HIV testing in children because, despite low overall ART coverage, children who are aware of their HIV status were highly likely to be on treatment.

## Supporting Information

Dataset S1 (CSV)
